# Supplementary material for: DNA replication initiation drives focal mutagenesis and rearrangements in human cancers
Source: Nat Commun. 2024 Dec 30;15:10850. doi: 10.1038/s41467-024-55148-3 (PMC11685606; doi:10.1038/s41467-024-55148-3)
Supplement: Supplementary file 1 — Supplementary Information [file 41467_2024_55148_MOESM1_ESM.pdf]

Supplementary Information

for

DNA replication initiation drives focal mutagenesis and rearrangements in human cancers

Pierre Murat[1,2,*], Guillaume Guilbaud[1] and Julian E. Sale[1,*]

[1] Division of Protein & Nucleic Acid Chemistry, MRC Laboratory of Molecular Biology, Francis Crick Avenue, Cambridge, CB2 0QH, UK

[2] Current address: Wellcome Sanger Institute, Hinxton, CB10 1RQ, UK

Corresponding authors. pm23@sanger.ac.uk (P.M.) and jes@mrc-lmb.cam.ac.uk (J.E.S.)

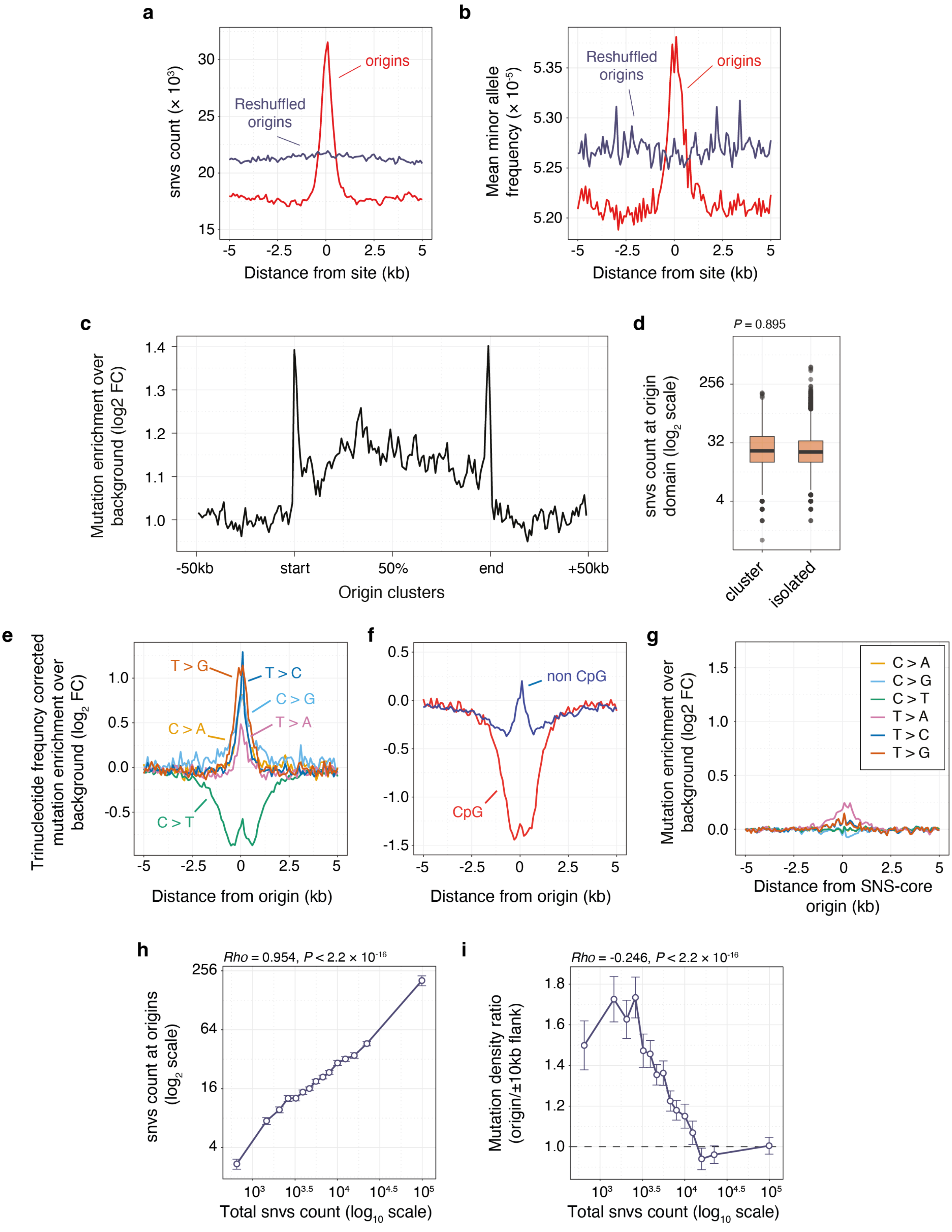

Supplementary Figure 1 | Mutational burden at constitutive origins. **a**, Count of single nucleotide variants (SNVs) and (**b**) mean allele frequency of alternative alleles, computed in 100 nt windows, at constitutive origins (red line) compared to control genomic locations

obtained through the reshuffling of origin coordinates (blue line), from aggregated pan-cancer ICGC mutation data. **c**, Mutation enrichment over background at origin clusters. Origins clusters were defined as genomic intervals larger than 20-kb with at least two constitutive origins. Cluster edges are defined by the extreme origins, hence the spikes in mutation at the boundaries of origin clusters. **d**, SNVs count at origin domains for isolated origins or origins within origin clusters. Box plots depict medians and interquartile ranges, with individual cancer samples represented as circles. The reported *P* value was computed using a Kolmogorov-Smirnov test. **e**, Pyrimidine mutation rates adjusted for trinucleotide frequency at constitute origins. **f**, C > T transition rates in CpG or non-CpG context at constitutive origins. **g**, Mutation rates associated with the six pyrimidine substitutions at 'core' SNS-seq origins corrected for local variation in base composition and background values (see the **Online Methods** section for detailed information). **h**, SNV count at origins and (**i**) the ratio of mutation counts within origin domains over those in adjacent flanking regions relative to the total (genome-wide) SNV count for cancer samples with more than 5,000 called mutations. Reported values represent the means and standard errors of the mean.

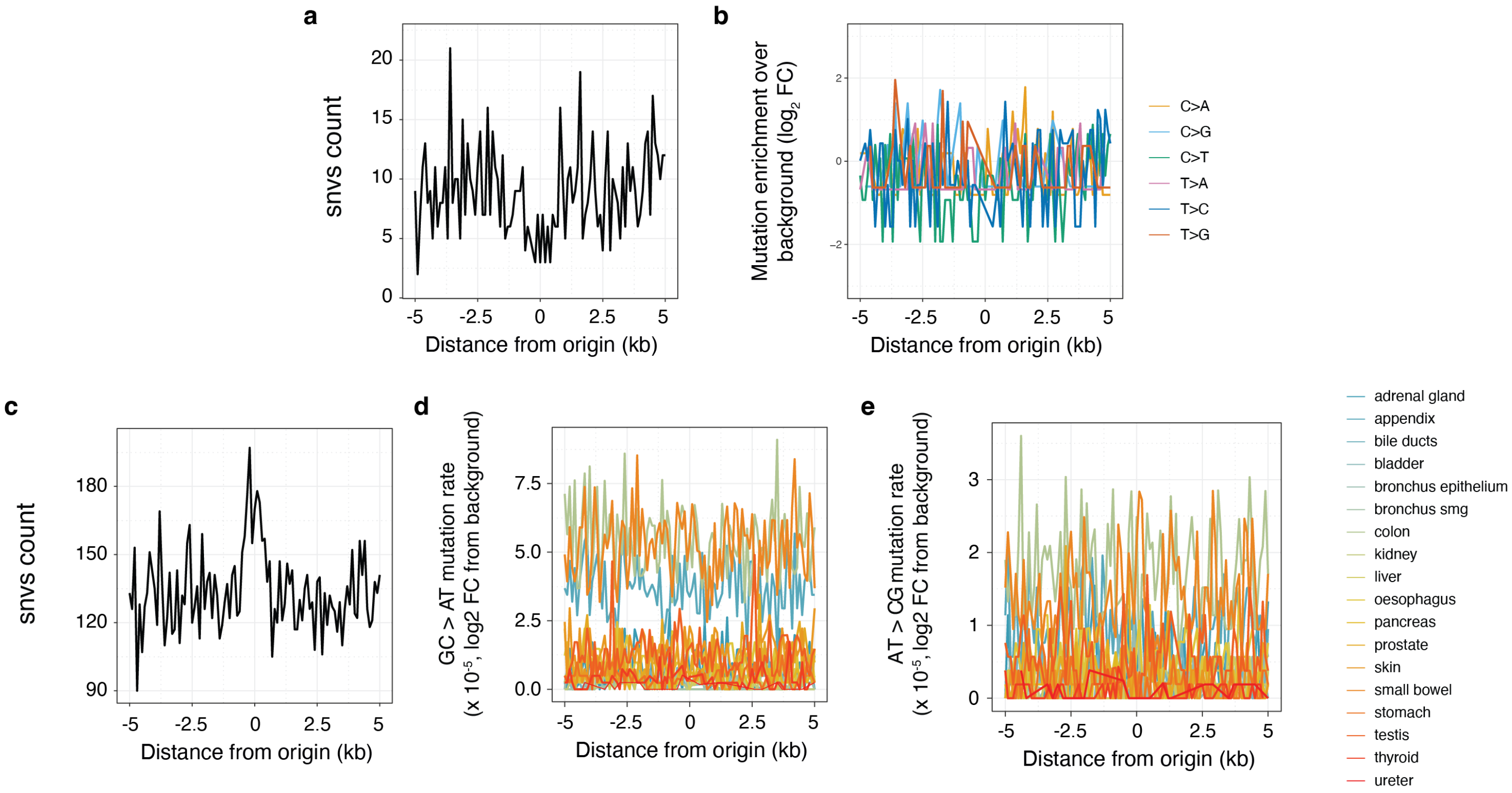

Supplementary Figure 2 | Origin mutagenesis in non-cancerous somatic tissues. a, Distribution of somatic SNVs mapped by Nanorate sequencing (NanoSeq), a duplex sequencing method with error rates below five errors per billion base pairs in single DNA molecules, from non-dividing cell populations. These include cord blood granulocytes, smooth cardiac muscle, post-mitotic neurons, colonic crypts, and sperm cells, at constitutive origins. [15] **b**, Corresponding mutation rates associated with the six pyrimidine substitutions at constitutive origins. Mutation rates are adjusted for local variation in base composition and background values. **c**, Distribution of SNVs aggregated from 18 somatic cell types, using multiple samples from the same individuals. [16] **d**, Corresponding GC > AT and (**e**) AT > GC mutation rates at constitutive origins. Mutation rates are adjusted for local variation in base composition and background values.

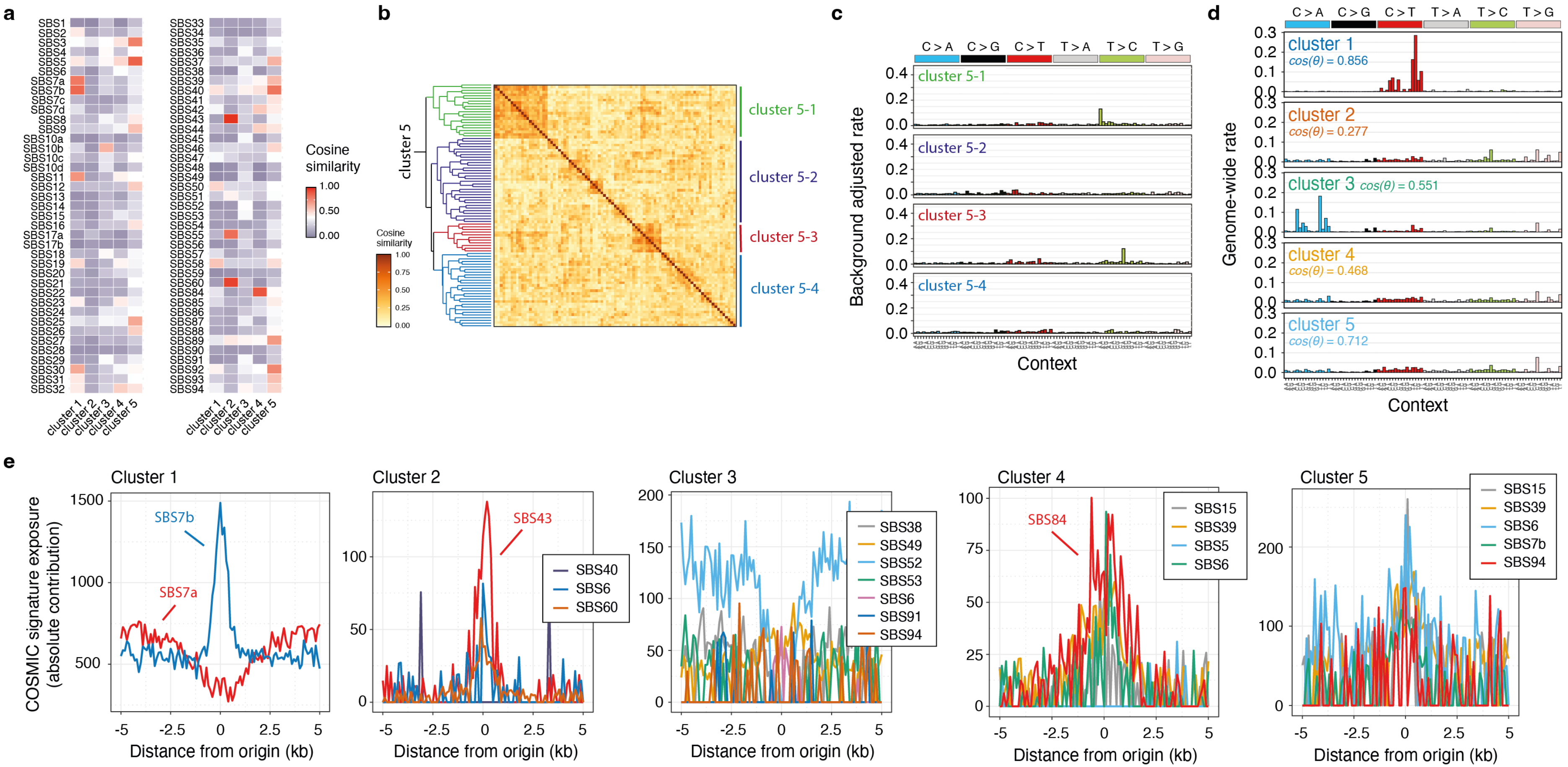

Supplementary Figure 3 | Characterisation of the mutational processes occurring at constitutive origins. a, Comparative analysis of origin-associated mutational signatures from tumours grouped into clusters 1 to 5, as defined in **Fig. 2a**, with established signatures of somatic mutations in human cancer tissues compiled by the Catalogue of Somatic Mutations in Cancer (COSMIC, release v3.2). The similarity between signatures is evaluated using cosine similarities. **b**, Cluster 5 was further divided into 4 sub-clusters; however (**c**) the mutational signatures associated with each sub-cluster are either flat or lack information. **d**, Genome-wide mutational signatures associated with tumours from clusters 1 to 5. Apart

from cluster 1, origin-associated signatures display minimal resemblance to their respective genome-wide signatures, as indicated by the reported cosine similarities. **e**, Absolute contribution of COSMIC signatures to mutagenesis at origins, computed in 100 nt windows, for tumours in clusters 1 to 5. Signature contributions were computed using an iterative fitting procedure to exclude signatures with minimal contribution. Only signatures that contribute to more than 50 mutations within at least one 100 nt window are reported.

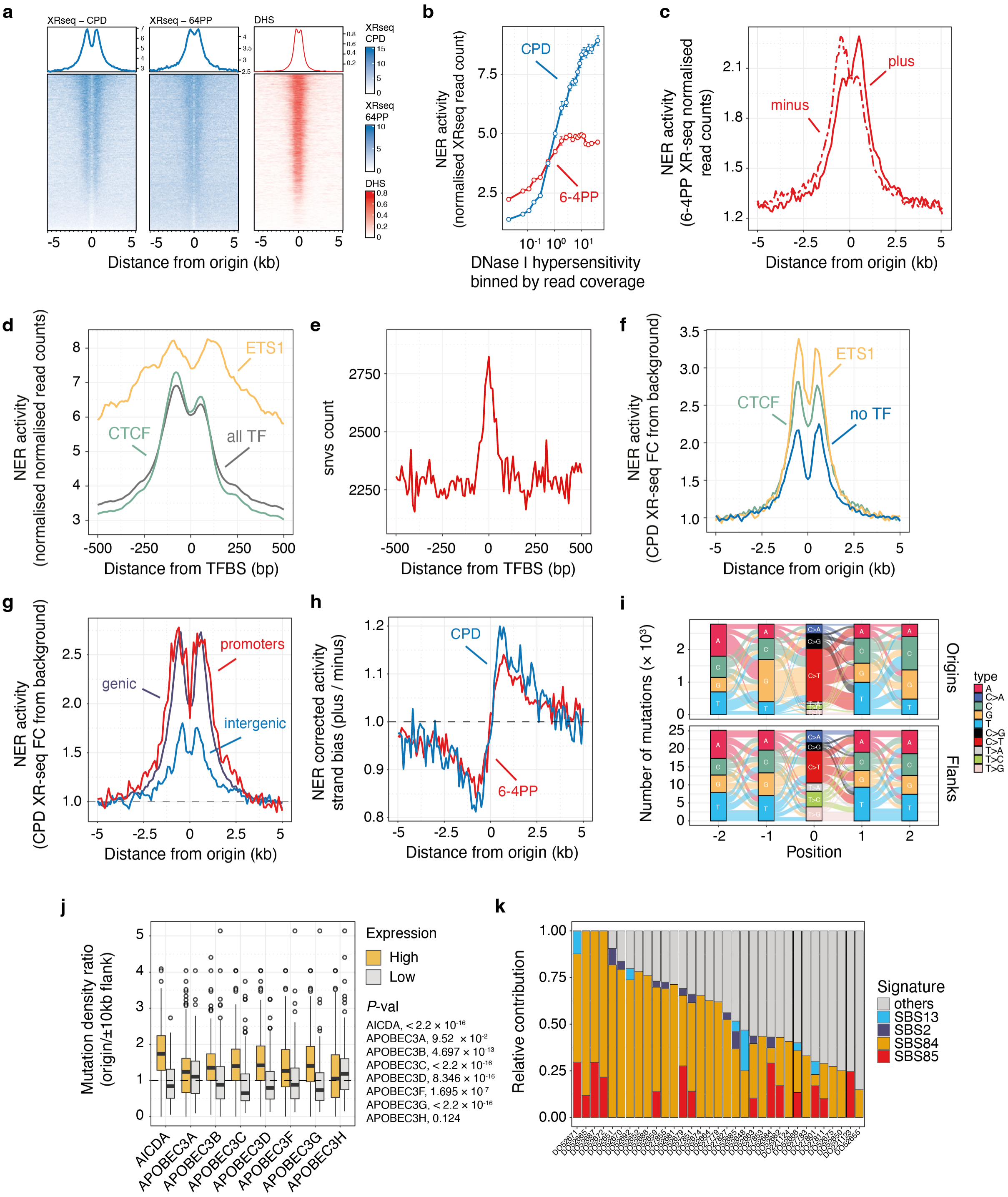

Supplementary Figure 4 | NER deficiency and deaminase activity at constitutive origins. **a**, Heatmaps displaying the coverage of XR-seq signals for CPD and 6-4 PP adducts (depicted in blue) and DNase I hypersensitivity (DHS, shown in red) at constitutive origins. The heatmaps are arranged based on decreasing CPD XR-seq signal values. CPD and 6-4 PP XR-seq signals are derived from ultraviolet-irradiated CSB/ERCC6 mutant NHF1 skin fibroblasts, while the DHS signal is from the GM04504 skin fibroblast cell line. **b**, Evaluation of NER activity, quantified by the normalised read count of XR-seq signals for CPD and 6-4 PP

adducts, in relation to origin accessibility, assessed by DHS read coverage, at origin domains (origin midpoints ± 1 kb). DHS signal is binned by read coverage, and NER activity represents the mean and standard errors to the mean of XR-seq read counts for each DHS bin. **c**, Strand-resolved XR-seq profiles for 6-4 PP at constitutive origins. **d**, NER activity and (**e**) SNV count at experimentally derived transcription factor binding sites (TFBSs). The TFBSs included in this analysis comprise binding sites for CTCF, ETS1, REST, EZH2, and NANOG in the GM23338 cell line. **f**, XR-seq profiles for CPD at constitutive origins with or without known binding sites for CTCF, ETS1, or any of the aforementioned transcription factors. **g**, XR-seq profiles for CPD at constitutive origins stratified by genomic location. **h**, NER-corrected activity strand bias computed from strand-resolved XR-seq signals for CPD and 6-4 PP in 100 bp windows. XR-seq signals were adjusted for dinucleotide base composition (refer to Online Methods). **i**, River plot illustrating the expanded contexts of mutations mapped within origin or origin-flanking domains for tumours of cluster 4. **j**, Mutation density ratios for individual B cell malignant lymphoma (MALY) samples, organised by the level of AICDA (AID) or APOBEC3s activity. High (depicted by orange boxes) and low (depicted by grey boxes) expression levels correspond to the higher and lower tertiles of gene expression, respectively. Box plots depict medians and interquartile ranges, with individual cancer samples represented as circles. The reported P values compare values for high versus low expression levels using Kolmogorov-Smirnov tests. **k**, Relative contribution of COSMIC signatures on mutagenesis within origin domains of MALY samples with over 50 SNVs at origin domains. Specifically, SBS2 and SBS13 are associated with the activity of the APOBEC family of cytidine deaminases, while SBS84 and SBS85 are associated with the direct or indirect activity of AID. This analysis shows that AID is the main deaminase operating at constitutive origins.

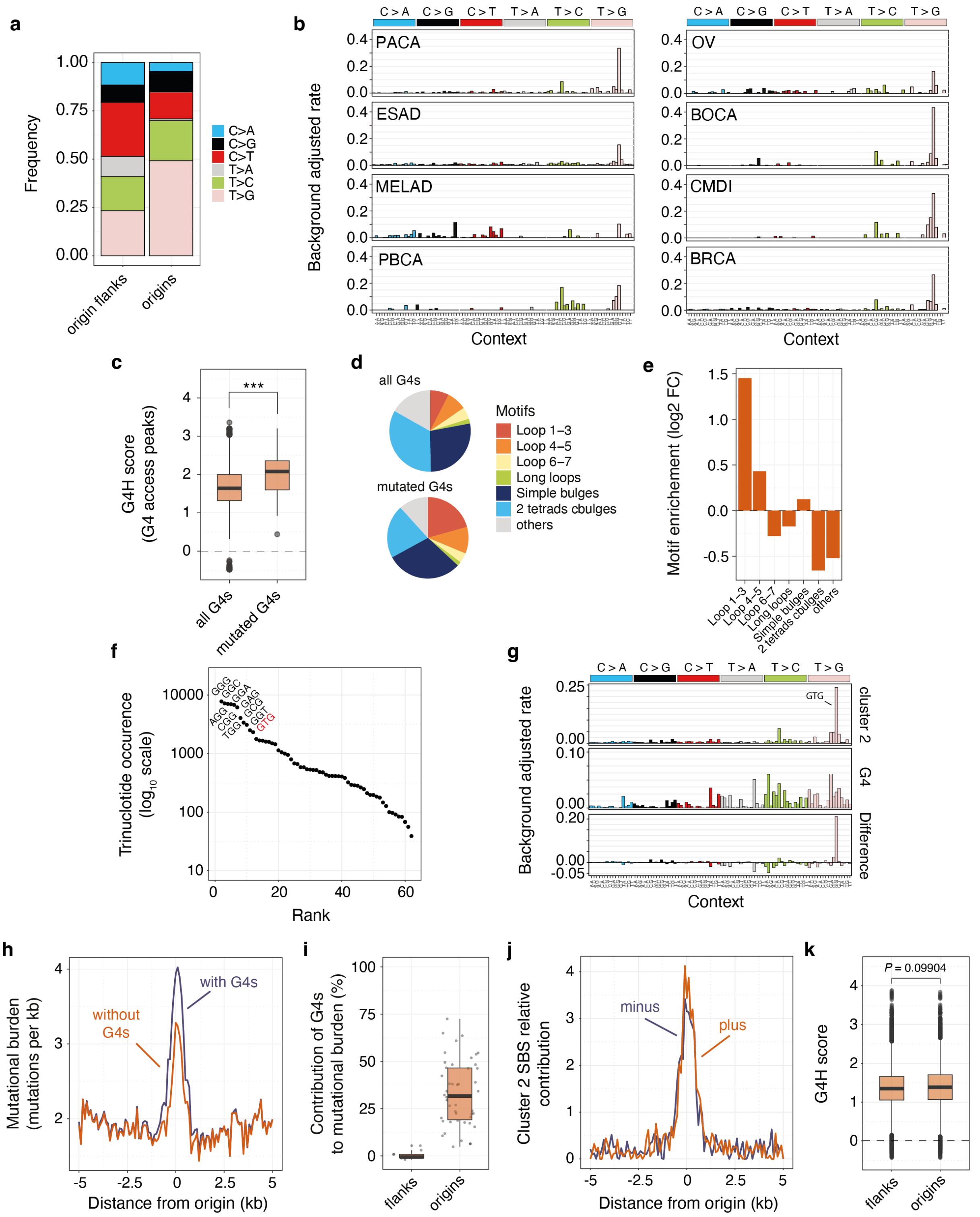

Supplementary Figure 5 | G-quadruplex mutational signature. a, Frequency distribution of each of the six pyrimidine substitutions within origin or origin-flanking domains of tumours from cluster 2, as defined in **Fig. 2a**. **b**, Origin-associated mutational signatures for cluster 2 tumours stratified by cancer types. Signatures were computed from aggregated mutation calls at origin domains for cluster 2 cancer samples and corrected as previously described. **c**,

Predicted stability (G4H score) of experimentally validated G4 structures by the G4access methods at origins. **d**, Distribution and (**e**) enrichment of G4 subtypes at origins based on whether they are mutated or not**.** Subtype categories are 'loop size' 1–3, 4–5 and 6–7, sequences with at least one loop of the respective length; simple bulge, sequences with a G4 with a bulge of 1–7 bases in one G-run or multiple 1-base bulges, 2-tetrads/complex bulge: sequences with a G4s with two G-bases per G-run or several bulges of 1–5 bases and other, other G4 types that do not fall into the former categories**. f**, Trinucleotide occurrences within G-quadruplex (G4) forming sequences conforming to the pattern $N_5G_{3+}N_{1–12}G_{3+}N_{1–12}G_{3+}N_{1–12}G_{3+}N_5$ (where N represents any base). The ten most recurrent trinucleotides are presented, with the characteristic GTG context highlighted in red. **g**, Background-adjusted mutational signatures extracted from origin mutations of cluster 2 or computed at G4 forming sequences corrected for G4 trinucleotide composition. The difference spectra underscore the overrepresentation of the GTG context. **h**, Mutational burden at origins with or without considering G4-forming sequences defined by the previous pattern. This plot shows average values across all cancer samples from cluster 2. **i**, Contribution of G4s to mutagenesis in individual cluster 2 cancer samples at origin domains or flanks. The contribution was calculated as the percentage of mutations occurring within a G4 structure matching the previous pattern. **j**, Strand-resolved contribution of cluster 2 mutational signatures to mutagenesis at the origins of cluster 2 tumours. Signature contribution was calculated by aggregating mutation calls from cluster 2 tumours. **k**, Distribution of G4 propensity scores, G4H scores, for G4 forming sequences conforming to the previous pattern found at origin or origin-flanking domains. Box plots depict medians and interquartile ranges, with outliers shown as grey dots. *P* value obtained from a Kolmogorov-Smirnov test.

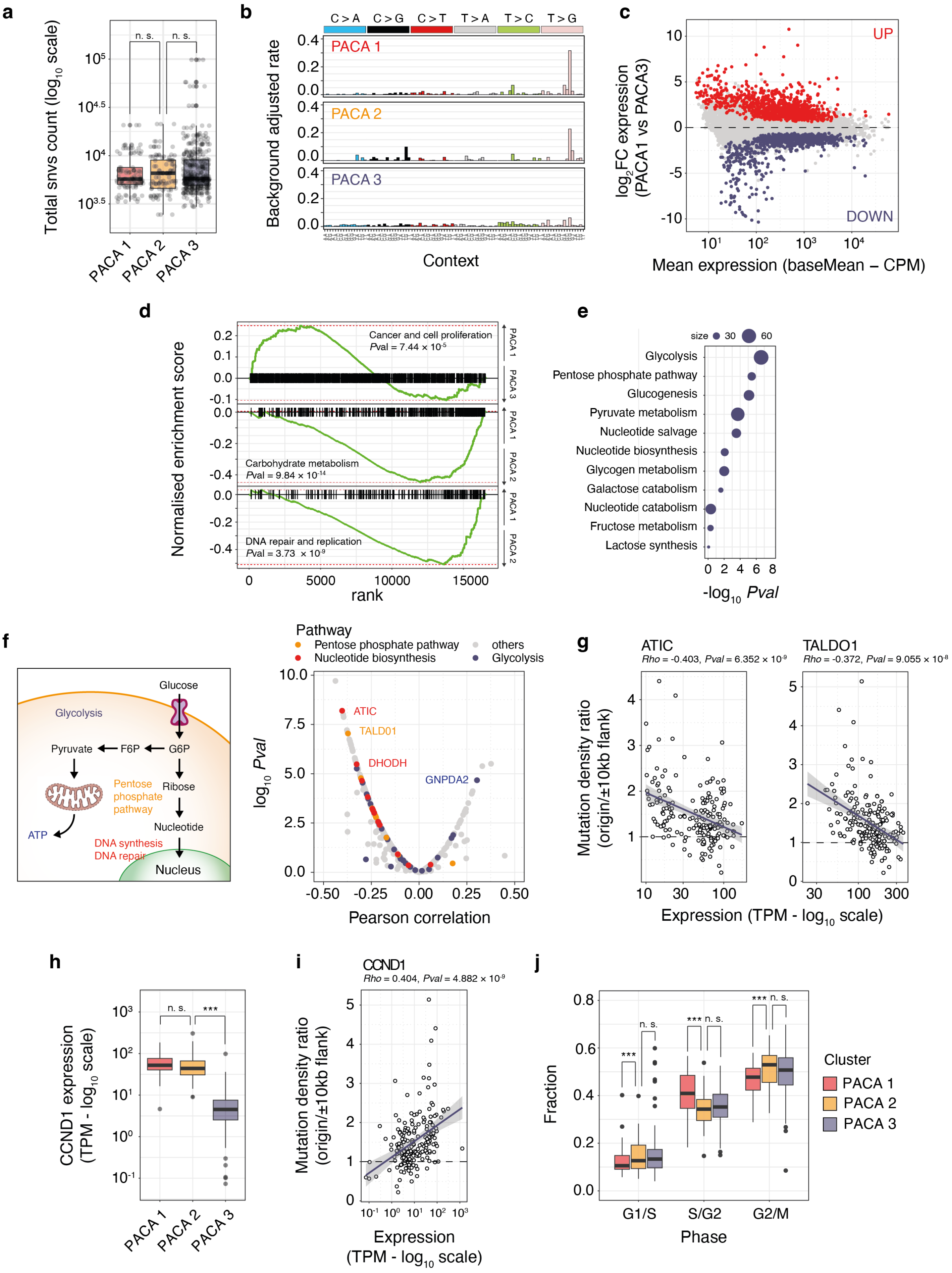

Supplementary Figure 6 | Determinants of origin mutagenesis. a, Distribution of genome-wide SNV counts for individual cancer samples categorised by PACA subtypes, as defined in **Fig. 5a**. **b**, Background adjusted mutational signatures at constitutive origin domains of PACA subtypes. **c**, DESeq2 differential gene expression analysis result contrasting gene expression in

PACA subtype 1 versus 3. The plot reports differences in gene expression, expressed in fold change, in function of basal level of gene expression. The top 1,000 genes up- and down-regulated genes are reported in red and blue respectively. **d**, Gene set enrichment analysis associated with the primary pathways depicted in **Fig. 5d**. Enrichment scores were computed based on compiled gene lists related to Cancer and cell proliferation (top), Carbohydrate metabolism (middle), and DNA repair and replication (bottom). *P* values were derived by combining the *P* values associated with sub-pathways obtained from permutation tests and using Fisher's method. **e**, Examination of Carbohydrate metabolism pathways. Primary KEGG pathways were dissected into smaller components to pinpoint specific processes exhibiting downregulation in PACA subtype 1 compared to subtypes 2 and 3. This analysis uncovered the dysregulation of Glycolysis and the Pentose Phosphate Pathway (PPP) in cluster 1 of PACA tumours. **f**. Schematic depiction (left panel) of the interconnection between the Glycolysis and PPP pathways. Glucose-6-phosphate acts as a common substrate for both pathways, contributing to pyruvate and ATP production in the Glycolysis pathway or ribonucleotide synthesis in the PPP. Thus, dysregulation of the PPP is anticipated to impact DNA synthesis and repair, resulting in increased mutagenesis at constitutive origins. To examine this hypothesis, we evaluated the Pearson correlation between the mutation ratio at origins versus origin flanks and the gene expression of enzymes involved in Carbohydrate metabolism (right panel). Enzymes associated with Glycolysis (blue), the PPP (orange), and Nucleotide biosynthesis (red) are highlighted. This analysis indicates that the expression of enzymes involved in the PPP and Nucleotide biosynthesis tends to negatively correlate with mutational burden at origins, underscoring a connection between glucose metabolism and mutagenesis at origins in pancreatic cancers. **g**, Negative correlations between the expression of key enzymes and mutational burden at origins is exemplified. ATIC is a bifunctional enzyme participating in the final two steps of purine biosynthesis, while TALDO1 is an enzyme responsible for generating ribose-5-phosphate. **h**, Expression levels of cyclin D1 in PACA tumours categorised in TPM. **i**, Cyclin D1 expression in TPM is function of the density ratio of mutations at origins to origin flanks for PACA samples. This analysis underscores the correlation between cell proliferation and mutational burden at origins. **j**, Fraction of cells in the G1/S, S/G2, and G2/M phases of the cell cycle for PACA tumours classified by subtypes. Cell cycle fractionation was inferred from the expression profiles of the four cyclin classes (refer to **Online Methods**): G1/S phase (computed as the ratio of cyclin E expression to the total cyclin expression), S/G2 phase (computed as the ratio of cyclin A expression to the total cyclin expression), and G2/M phase (computed as the ratio of cyclin B expression to the total cyclin expression). In panels **a**, **h**, and

j, box plots illustrate medians and interquartile ranges, while individual cancer samples are depicted as grey dots. *n. s.* non-significant, ***$P < 0.001$, Kolmogorov-Smirnov test.

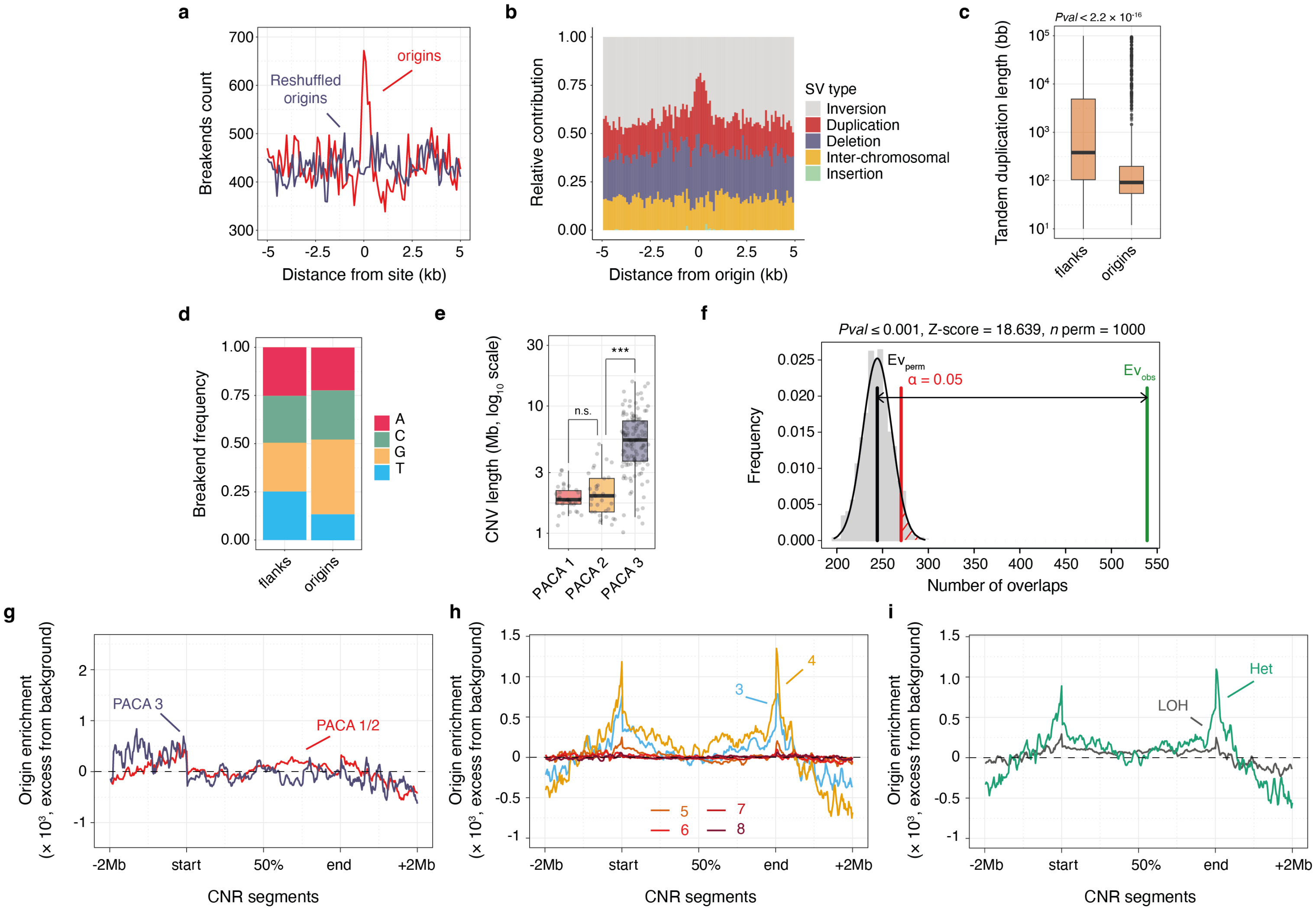

Supplementary Figure 7 | Constitutive origins are hotspots for genome rearrangements. a, Structural variants (SVs) breakends counts, computed in 100 nt windows, at constitutive origins (red line) compared to control genomic locations obtained through the reshuffling of origin

coordinates (blue line), using aggregated pan-cancer ICGC mutation data. **b**, Relative contribution of SV type at constitutive origins. **c**, Distribution of the length of tandem duplication events mapped at origin or origin flanking domains. Box plots present medians and interquartile ranges. The *P* value is derived from a Kolmogorov-Smirnov test. **d**, Nucleotide frequency at SV breakends mapped at origin or origin flanking domains. **e**, Distribution of copy number variants (CNVs) length for individual cancer samples categorised by PACA subtypes, as defined in **Fig. 5a**. Box plots report medians and interquartile ranges, with individual cancer samples depicted as grey dots. *n. s.* non-significant, ***$P < 0.001$, Kolmogorov-Smirnov test. **f**, Permutation test result assessing the enrichment of CNV breakpoints at constitutive origin domains. 1,000 permutations were performed, and the plot reports the distribution of randomised and observed numbers of overlaps. Enrichment of origins within CNV segments mapped in PACA subtypes considering (**g**) copy neutral and loss segments (copy number ≤ 2), (**h**) amplified CNV segments of different copy numbers and (**i**) amplified CNV segments of 4 copy numbers displaying either sign of loss of heterozygosity (LOH) or not (Het). Enrichment values were evaluated by partitioning CNV segments and their flanking domains (± 2 Mb) into an equal number of windows, calculating the number of origins per segment and per window, and then determining the mean number of origins per window. This value was then adjusted by subtracting the mean values observed in segment flanking domains. The resulting origin enrichment value indicates the excess of origins within a given window.
