# Supplementary material for: DNA replication initiation drives focal mutagenesis and rearrangements in human cancers
Source: Nat Commun. 2024 Dec 30;15:10850. doi: 10.1038/s41467-024-55148-3 (PMC11685606; doi:10.1038/s41467-024-55148-3)
Supplement: Supplementary file 2 — Description of Additional Supplementary Files [file 41467_2024_55148_MOESM2_ESM.pdf]

Description of Additional Supplementary Files

File Name: Supplementary Data 1

Description: BED file containing the positions of constitutive replication origins lifted over from hg38 to hg19, along with their calculated efficiency (See Reference 6).
